# Supplementary material for: Comparative multiomics analysis of cell physiological state after culture in a basket bioreactor
Source: Sci Rep. 2022 Nov 23;12:20161. doi: 10.1038/s41598-022-24687-4 (PMC9686226; doi:10.1038/s41598-022-24687-4)
Supplement: Supplementary file 1 — Supplementary Information 1. [file 41598_2022_24687_MOESM1_ESM.zip › raw data/Transcriptomic raw data/6.Enrichment/2.KEGG/TE1vsTC1/GSEA/HUNTINGTON_DISEASE(CSAB05016).html]

Details for gene set HUNTINGTON\_DISEASE(CSAB05016)[GSEA]

|  || Dataset | readcount.sample |
| Phenotype | sample.cls |
| Upregulated in class | TC1 |
| GeneSet | HUNTINGTON\_DISEASE(CSAB05016) |
| Enrichment Score (ES) | -0.39919627 |
| Normalized Enrichment Score (NES) | -0.96799713 |
| Nominal p-value | 0.48773006 |
| FDR q-value | 0.9065701 |
| FWER p-Value | 1.0 |
Table: GSEA Results Summary

  

Fig 1: Enrichment plot: HUNTINGTON\_DISEASE(CSAB05016)      
 Profile of the Running ES Score & Positions of GeneSet Members on the Rank Ordered List

  

| PROBE | DESCRIPTION (from dataset) | GENE SYMBOL | GENE\_TITLE | RANK IN GENE LIST | RANK METRIC SCORE | RUNNING ES | CORE ENRICHMENT || 1 | novel.518 | - |  |  | 8940 | 1.089 | -0.2040 | Yes |
| 2 | novel.517 | - |  |  | 11208 | 0.944 | -0.1362 | Yes |
| 3 | novel.514 | - |  |  | 14470 | 0.741 | -0.1490 | Yes |
| 4 | novel.471 | - |  |  | 15225 | 0.693 | -0.0585 | Yes |
| 5 | novel.529 | - |  |  | 15745 | 0.658 | 0.0363 | Yes |
| 6 | novel.530 | - |  |  | 16765 | 0.586 | 0.0958 | Yes |
| 7 | novel.574 | - |  |  | 18341 | 0.456 | 0.1072 | Yes |
| 8 | novel.516 | - |  |  | 18769 | 0.414 | 0.1622 | Yes |
Table: GSEA details [plain text format]

  

Fig 2: HUNTINGTON\_DISEASE(CSAB05016)      
 Blue-Pink O' Gram in the Space of the Analyzed GeneSet

  

Fig 3: HUNTINGTON\_DISEASE(CSAB05016): Random ES distribution      
 Gene set null distribution of ES for **HUNTINGTON\_DISEASE(CSAB05016)**
